# The gram-negative bacterium *Escherichia coli* as a model for testing the effect of carbonic anhydrase inhibition on bacterial growth

**DOI:** 10.1080/14756366.2022.2101644

**Published:** 2022-07-28

**Authors:** Viviana De Luca, Vincenzo Carginale, Claudiu T. Supuran, Clemente Capasso

**Affiliations:** a Institute of Biosciences and Bioresources, National Research Council, Napoli, Italy; b Section of Pharmaceutical and Nutraceutical Sciences, Department of Neurofarba, University of Florence, Florence, Italy

**Keywords:** Carbonic anhydrases, sulphonamide, inhibitor, phenol-sulphuric acid assay, glucose consumption, bacterial growth, microorganism lifecycle

## Abstract

Carbonic anhydrases, catalysing the reversible CO_2_ hydration reaction, contribute in all living organisms to the maintenance of stable metabolic functions depending on intracellular concentrations of carbon dioxide, bicarbonate, and protons. Recent studies have examined how CAs affect bacterial lifecycle, considering these enzymes druggable targets due to interference with their activities by using inhibitors or activators. Here, we propose *Escherichia coli* cells as a model for testing the effect of acetazolamide (AZA), a potent CA inhibitor, on bacterial survival by evaluating *E. coli* growth through its glucose consumption. AZA, at concentrations higher than 31.2 µg/mL, was able to impair *E. coli* growth and glucose uptake. AZA is a good inhibitor of the two recombinant *E. coli* CAs, the β-CA CynT2, and the γ-CA EcoCAγ, with KIs of 227 and 248 nM, respectively. This study provides a proof-of-concept, low-cost method for identifying effective CA inhibitors capable of impairing bacterial metabolism.

## 1. Introduction

Over the past few years, the knowledge of bacterial carbonic anhydrases (CAs, EC 4.2.1.1) has significantly increased. Many reports showed that these metalloenzymes catalyse the physiologically crucial reversible CO_2_ hydration reaction (CO_2_ + H_2_O ⇋ HCO_3_^–^ + H^+^) with high catalytic rates (k_cat_) ranging from 10^4^ to 10^6^ s^−1^_._^1^ It is thus readily apparent that CAs help all living organisms in balancing the endogenous levels of carbon dioxide (CO_2_), bicarbonate (HCO_3_^–^), and protons (H^+^), which are essential for sustaining a range of metabolic activities^2–6^. The non-catalytically occurring CO_2_ hydration reaction cannot provide bicarbonate to support/bicarbonate to support the central metabolism rates, since the k*_cat hydration_* of 0.15 s^−1^ and k*_cat dehydration_* of 50.0 s^−1^ are too low at physiological pH to satisfy the organism's metabolic demands^7^. Because of their central role in catalysing critical reactions in metabolic pathways, these enzymes have ultimately been considered as attractive druggable targets whose inhibition might impair the microorganism lifecycle^6–10^. In all life kingdoms, eight CA classes have been identified so far, and they are indicated with the Greek letters α, β, γ, δ, ζ, η, θ, and ι^7^^,^^9–12^. Up to date, four CA-classes (α, β, γ, and ι) have been identified in bacteria^2–6^. In our previous papers and at the time when only three CA-classes were known to be present in bacteria (α, β, and γ), we allocated them to Gram-negative bacterial compartments (periplasmic space and cytoplasm), considering their amino acid sequence features^7^^,^^9^^,^^11^^,^^12^.

**Figure 1.**
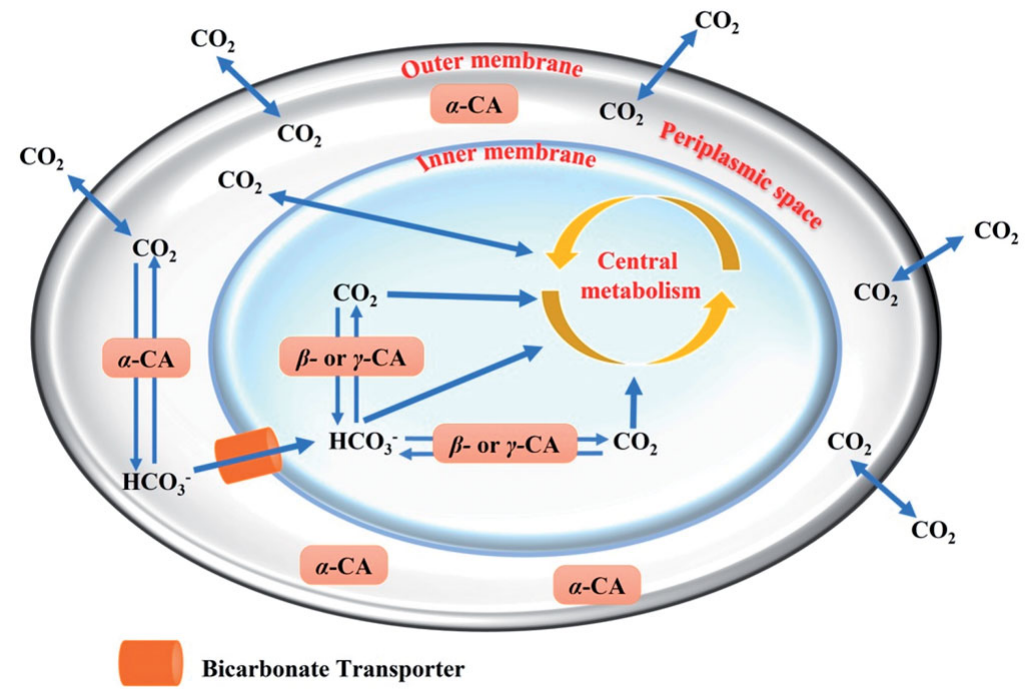
Schematic representation of a gram-negative bacterium with the inner and outer membranes delimiting the periplasmic (in white) and cellular cytoplasm (in light blue). The passive CO_2_ diffusion through the bacterial cell and the bicarbonate transporter (orange colour), which actively vehicles bicarbonate from the periplasmic space to the cytoplasm are shown together with various CAs and their putative role(s).

As shown in Figure 1, we proposed that α-CAs, characterised by a signal peptide at the *N*-terminus of the polypeptide chain, reside in the periplasmic region and have the function of avoiding the loss of CO_2_ through diffusion from the bacteria cells, converting it into bicarbonate, which is transported inside the cytoplasm by bicarbonate transporters^7^. In contrast, the β- or γ-classes which are localised in the cytoplasm, perform intracellular functions, such as CO_2_/HCO_3_^–^ transport/homeostasis and pH regulation^7^. Recently, in some Gram-negative bacteria lacking α-CAs, it has been discovered the presence of β-, γ-, and ι-CAs with a potential signal peptide at their N-terminus, which probably may have a function similar to that performed by α-CAs in the species where this class is present^10^^,^^11^^,^^13–16^. The function of CAs in bacteria and how these enzymes influence the bacterial lifecycle have been recently examined in the literature. For example, the β-CA encoded in the genome of *Ralstonia eutropha* is essential for its growth at ambient CO_2_ concentrations.^17^ In *Escherichia coli*, the β-CA (CynT) produces the HCO_3_^-^, preventing bacteria from running out of bicarbonate due to the breakdown of cyanate as well as other metabolic processes. A second *E. coli* β-CA isoform (CynT2) was shown to be critical for bacterial proliferation at atmospheric CO_2_ concentrations^18^^,^^19^. Intriguingly, multiple examples of correlations between CA activity and the ability of bacteria, such as *Vibrio cholerae*^20^*, Mycobacterium tuberculosis*^21–25^*, Salmonella enterica*^26–28^, *Pseudomonas aeruginosa*^29^, *Helicobacter pylori*^30–32^, etc., to persist, become pathogenic and cause disease in humans can be found in the literature. Thus, it is readily evident that the inhibition of bacterial CA may decrease the pathogen survival/fitness. Fortunately, many specific chemical classes of CA inhibitors (CAIs) were reported in the scientific literature^33–36^. Recently, several FDA-approved CAIs were modified to target vancomycin-resistant enterococci (VRE)^37^. Ethoxzolamide (EZA), an authorised diuretic and CAI, was shown to kill *Helicobacter pylori in vitro*, suggesting it could be turned into an anti-*H. pylori* medication^38^. Finally, acetazolamide inhibited the growth of the Gram-negative bacterium *Neisseria gonorrhoeae* both *in vitro* as well as in *in vivo* mouse models of gonococcal genital tract infection^39^.

In the present work, we propose the use of *Escherichia coli* cells, which can be easily manipulated in the lab, as a model for testing the effect of CAIs on the bacterial lifecycle. We used as example of CAI acetazolamide (AZA), a potent CA inhibitor, and evaluated *E. coli* growth as well as its glucose consumption. Glucose was added as the only carbon source to a liquid minimal culture media for bacterial growth. It has been demonstrated earlier by our groups that AZA was able to inhibit *in vitro* the two recombinant *E. coli* CAs, β-CA (CynT2) and γ-CA (EcoCAγ), with K_I_s of 227 and 248 nM, respectively^40^^,^^41^.

The present work proposes a proof-of-concept, inexpensive method for selecting CAIs able to impair bacterial metabolism, which could be employed in future applications in the fight against pathogenic bacteria, which are difficult to be managed in normal laboratories without the appropriate biosafety precautions.

## 2. Materials and methods

### 2.1. Cloning, expression, and purification of the recombinant E.coli β- and γ-CAs

The synthetic genes encoding for the *E.coli* β- and γ-CA were synthesised by the Invitrogen GeneArt (ThermoFisher Scientific), a company specialised in gene synthesis. The genes were cloned into the expression vector pET100D-Topo and heterologously expressed as described previously^40–43^. The recovered *E. coli* β- and γ-CAs were >90% pure. The protein quantification was carried out by the Bradford method (BioRAD)^44^.

### 2.2. Protonography

Following the electrophoresis, the SDS-PAGE gel was subject to protonography to detect the yellows bands due to the CO_2_ hydratase activity. The protonogram was developed as previously described by our groups^15^^,^^45^^,^^46^.

### 2.3. Stopped flow CA assay

The CO_2_ hydratase activity of the soluble recombinant enzymes, the corresponding kinetic constants, and AZA inhibition constants were determined using the stopped-flow technique as previously described^41^.

### 2.4. E. Coli cells preparation

*E. coli* DH5α cells were streaked under sterile conditions on an LB Agar plate with no selection from a strain stock stored at −80 °C mixed with 60% (v/v) glycerol (final concentration 30% v/v). Plates were incubated overnight at 37 °C. The day after, a few *E. coli* single colonies were picked up with a sterile applicator stick and inoculated in a 250 ml flask containing 50 ml of LB medium under non-selective conditions and grown overnight at 37 °C with aeration (≥150 rpm). The cells from the overnight growth were harvested by centrifugation at 4 °C, centrifuged at 1500×*g* for 30 min, and suspended in fresh LB media to a final optical density (OD) of 0.15 at 600 nm and growth up to 0.6 OD and subsequently counted.

### 2.5. E. Coli growth in presence of AZA

Eight acetazolamide (AZA) concentrations were used to test the effect of this pharmacological inhibitor on bacterial lifecycle (4000, 2000, 1000, 500, 250, 125, 62.5, and 31.2 µg/mL). AZA was dissolved in 100% dimethyl sulfoxide (DMSO) at 20 mg/mL stock concentration. The highest final concentration of DMSO in the assay was 5% (this concentration has no effects on the bacterial growth). Cells were seeded in a 24 multi-well plate at a final concentration of 100,000 cells/mL, containing Mueller Hinton (MH) broth as a medium. Cells were grown in an incubator for 6 h at 37 °C. As a control, we used *E. coli* cells with no AZA added. Afterward, 1 ml of bacterial growth from each well was collected and OD was read at 600 nm using a Varian Cary 50 Scan UV Visible Spectrophotometer.

### 2.6. Effect of AZA on E. coli glucose uptake

AZA was tested at 4000, 2000, 1000, 500, 250, 125, 62.5, and 31.2 µg/mL for its effect on glucose uptake. AZA stock solution was prepared as described in paragraph 2.5. The assay highest DMSO concentration was 5%, which has no effects on bacterial glucose uptake, as demonstrated by incubating the cells in the presence of glucose and 5% DMSO. One hundred thousand cells/mL were seeded in a 24 multi-well plate containing minimal medium (M9) supplemented with glucose at a final concentration of 0.4% and incubated at 37 °C. Cells with no AZA were used as control. An aliquot of the sample (5 µL) was used to analyse the sugar concentration. It was diluted up to 200 µL with H_2_O, 1 mM EDTA and subjected to the Phenol Sulphuric Acid assay as described in the literature^47^. Briefly, to determine the glucose uptake, 200 µL of Phenol 5% and 1 ml of concentrated sulphuric acid were added to the sample aliquots to reach a final reaction volume of 1.4 ml. The reactions were let to stand for 15 min. Then, the sample was transferred to a cuvette at 485 nm using a Varian Cary 50 Scan UV Visible Spectrophotometer. The assay standard curve was performed with the following concentrations of glucose: 0, 2, 4, 8, 10, 12, and 14 µg/mL, prepared from a glucose 100 mg/L stock solution diluted in distilled water and 1 mM EDTA.

## 3. Results and discussion

### 3.1. Cynt2 (β-CA) and the EcoCAγ (γ-CA) production and characterisation

**Figure 2.**
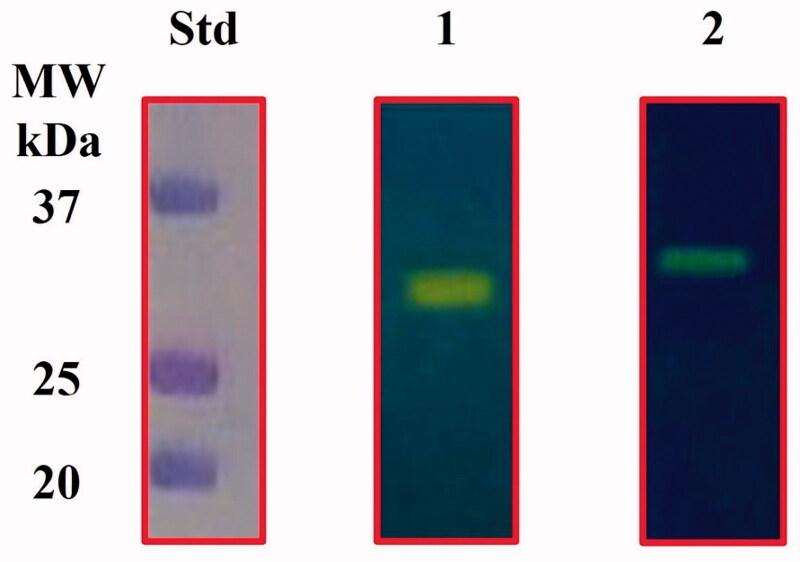
Protonogram of the two heterologously expressed *E. coli* CAs. The yellow band on the protonogram corresponds to the enzyme activity, which is responsible for the pH decrease from 8.2 to 6.8 due the dye transition point at acidic pH values. Legend: Lane STD, molecular markers; Lane 1, purified CynT2; Lane 2, purified EcoCAγ.

The *E. coli* genome encodes for β-, γ-, and ι-CAs. Our groups heterologously overexpressed the CynT2 (β-CA) and the EcoCAγ (γ-CA) as fusion proteins with six tandem histidines (His6-Tag) at the polypeptide chain N-terminus. A high degree of purity of both proteins was accomplished by isolating them from the soluble cytoplasmic protein fraction of the bacterial host cells with a nickel-charged resin that had a high affinity for the polyhistidine motif. Protonography of the two purified CAs is shown in Figure 2.

The catalytic activity of the recombinant enzymes is revealed by the yellow band due to the CO_2_ hydratase activity that correlated with the molecular weight of the CynT2 (29.0 kDa) and EcoCAγ (33.0 kDa) monomers. β- and γ-CAs are enzymes active as dimers and trimers, respectively. However, the yellow band appeared in the position of the monomeric forms of these enzymes, because SDS removal during the protonogram development leads to the rearrangement of the β- and γ-CAs monomers in the gel, which probably restore the active dimeric or tetrameric forms of the β-CA as well as the trimeric form of the γ-CA.

The stopped-flow technique was utilised to ascertain the CO_2_ hydratase activity as well as the respective kinetic constants of the purified enzymes. Therefore, we report these measurements in comparison with those obtained for the two human isoenzymes (CAI and CAII), previously purified in our labs^41^.

CynT2 and EcoCAγ resulted to be excellent catalysts for the CO_2_ hydration reaction (k_cat_ of 5.3 × 10^5^ s^−1^ and 5.7 × 10^5^ s^−1^, respectively) and were inhibited by acetazolamide (AAZ), a well-known pharmacological CA inhibitor, with a K_I_ of 227 nM and 248, respectively (Table 1).

**Table 1.** Kinetic characteristics of the CO_2_ hydration reaction catalysed by α-, β-, and γ-CA enzymes. Human (h) hCA I and II (α-CAs), at 20° C and pH 7.5 in 10 mM HEPES buffer; CynT2 and EcoCAγ determined at 20° C, pH 8.3 in 20 mM TRIS buffer and 20 mM NaClO_4_. We also present inhibition data using the clinically relevant sulphonamide AZA (5-acetamido-1,3,4-thiadiazole-2-sulphonamide).

Organism	Acronym	Class	k_cat_ (s^-1^)	k_cat_/K_m_ (M^-1^ x s^-1^)	K_I_ (acetazolamide) (nM)
*Homo sapiens*	hCA I	α	2.0 × 10^5^	5.0 × 10^7^	250
	hCA II	α	1.4 × 10^6^	1.5 × 10^8^	12
*Escherichia coli*	CynT2	β	5.3 × 10^5^	4.1 × 10^7^	227
	EcoCAγ	γ	5.7 × 10^5^	6.9 × 10^6^	248

Mean from 3 different assays by a stopped-flow technique (errors were in the range of ±5–10% of the reported values).

### 3.2. CAIs as antibacterials

The drug-approach method involves three critical points: *i*) finding out which metabolic pathways are necessary for the pathogen to live; *ii*) finding out the essential enzymes, which govern a specific metabolic pathway. The optimal approach would be to find target enzymes that are only found in the pathogen and not in the host. Even if, often this situation is uncommon since most important metabolic pathways are the same in all living things; *iii*) finding/designing small molecules and/or peptides that can selectively inhibit the target enzyme, and thus the pathogen growth. It is readily apparent that the two *E. coli* CAs meet these three points since CAs have a central role in catalysing critical reactions in bacterial metabolic pathways, such as the CO_2_/HCO_3_^-^ bacterial homeostasis. Besides, they can be effectively inhibited by many inhibitors, such as simple aromatic/heterocyclic sulphonamides, which are frequently used as building blocks in the development of novel effective, and selective families of such pharmaceutical drugs. Of course, the criterium *ii*) is respected only in part because CAs are ubiquitous enzymes. In this scenario, the limitation can be circumvented by developing and isolating compounds that selectively inhibit bacterial enzymes while leaving host protein homologs unaffected or only marginally inhibited. Trimethoprim, for example, was discovered to specifically block the bacterial enzyme dihydrofolate reductase (DHFR), but not the human DHFR.

The possibility of inhibiting bacterial CAs is remarkable, especially when one considers the emergence of drug resistance to the existing antimicrobial medications, which is one of the most severe problems the human population needs to face. Thus, understanding the inhibitory patterns of CAs from various bacterial species and identifying potent and selective inhibitors that can impair bacterial growth is essential.

#### 3.2.1. Effect of AZA on the bacterial growth

**Figure 3.**
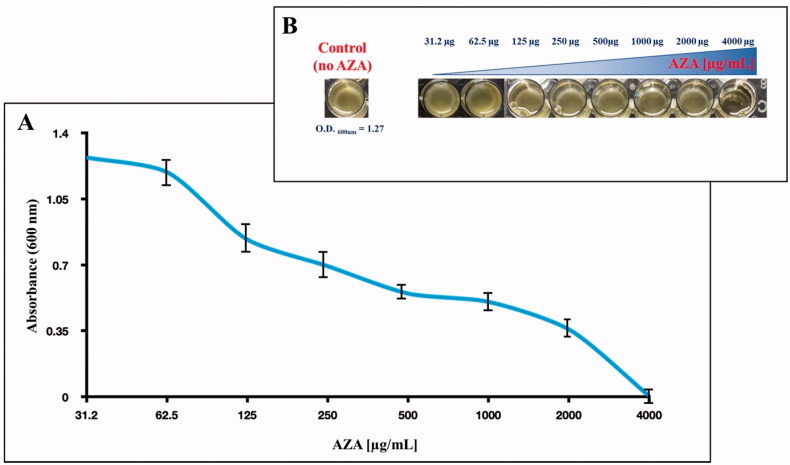
Effect of AZA on *E. coli* cells grown in MH for 6 h in presence of the eight different concentrations of AZA reported on the x-axis. (A) Bacterial growth has been monitored by measuring the optical density at 600 nm. (B) reports the wells showing the *E. coli* growth and the control represented by the bacterial cells with no AZA. Each data point is the mean value of at least three independent experiments.

In this context, we have tested the effect of acetazolamide (AZA), an FDA-approved effective sulphonamide CAI, on bacterial survival by evaluating *E. coli* growth. The growth of *E. coli* was monitored after 6 h incubation with the CAI, by measuring the OD at the wavelength of 600 nm, which is related to the cell number, and incubating 1.0 × 10^5^ *E. coli* cells in an MH medium containing different concentrations of AZA. As a control, we used *E. coli* cells with no AZA. The result is shown in Figure 3(A,B).

**Figure 4.**
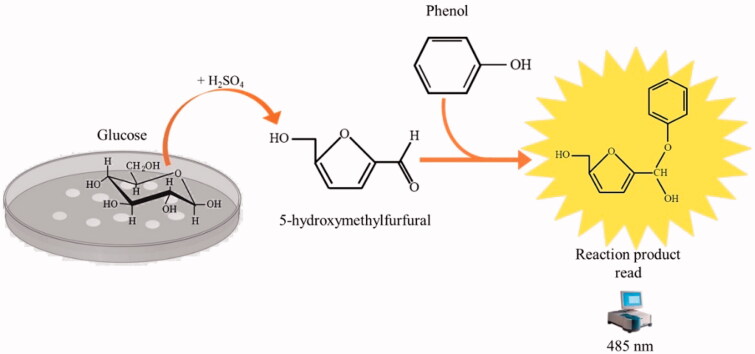
The reaction of phenol–sulphuric acid assay with glucose. Phenol in the presence of sulphuric acid is used for the quantitative colorimetric determination of glucose. Glucose by the action of concentrated sulphuric acid is dehydrated to hydroxymethylfurfural. These compounds then react with phenol to produce the reaction product (phenol- hydroxymethylfurfural), having a yellow-gold colour. The phenol-hydroxymethylfurfural is directly proportional to the amount of glucose present in the medium.

It is readily apparent that the increased AZA concentrations in the MH medium impair bacterial growth (Figure 3). As shown in Figure 3(A,B), the inhibiting effect of AZA on *E. coli* growth was maximal at 4000 µg/mL (absorbance 0.0025, and no bacterial cells were visible in the well, Panel B)). Lowering the AZA concentration (from 4000 to 31.2 µg/mL), the absorbance reached the value that corresponds to the control (OD600nm= 1.2) at the inhibitor concentrations in the range of 62.5 to 31.2 µg/mL (Figure 4), meaning that at these concentrations, the inhibitor did not affect cell growth.

#### 3.2.2. Effect of AZA on bacterial glucose uptake

Microbial metabolism relies on carbon sources, which are essential for biosynthetic activities and in many cases, energy metabolism. Besides, the rate at which carbon sources are used is closely related to metabolic activity. Glucose is arguably the most used and monitored carbohydrate, and it is quickly monitored by utilising the assays of the phenol–sulphuric acid (Figure 4)^47^.

**Figure 5.**
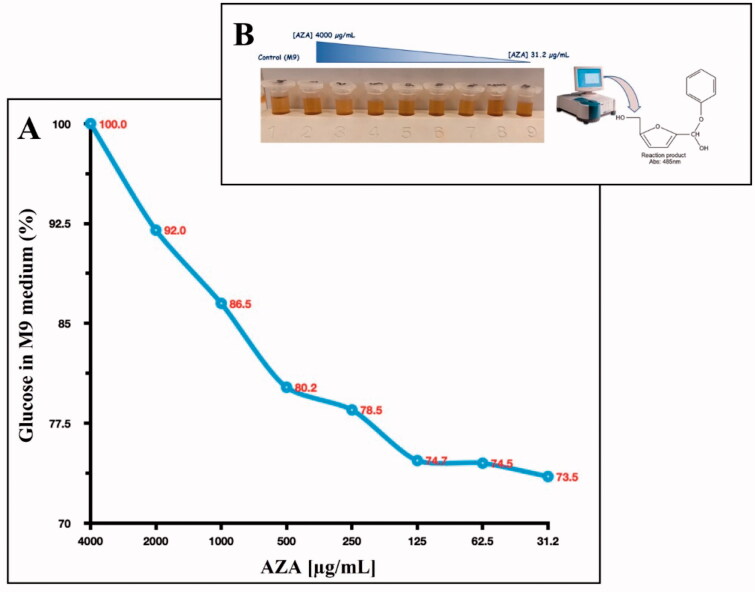
Glucose consumption by *E. coli* cells. Panel A shows the glucose consumption curve at different AZA concentrations (from 4000 to 31.2 g/mL) and using 1.0 × 10^5^ *E. coli* cells. The phenol–sulphuric acid assay was performed after the *E. coli* cells were incubated for 6 h in the M9 medium supplemented with a final concentration of 0.4% glucose. Panel B shows the tubes containing the different concentrations of AZA and the glucose at 04%. The yellow-gold colour was developed by the reaction of the phenol with the 5- hydroxymethylfurfural, which was produced by the interaction of sulphuric acid with glucose. Each data point is the mean value of at least three independent experiments.

Thus, we explored the glucose consumption by *E. coli* cells. The experiment was performed by adding glucose at a final concentration of 0.4% to a liquid minimal culture media (M9 medium) as the only carbon source. The M9 contained 1.0 × 10^5^ *E. coli* cells and different concentrations of AZA ranging from 4000 to 31.2 µg/mL (the same concentration used to monitor the bacterial growth). The glucose consumption was detected after 6 h by prevailing an aliquoted of the M9 containing glucose and measuring the phenol-hydroxymethylfurfural optical density at 485 nm (see Figure 5). As a control, we used the M9 with 0.4% glucose. As shown in Figure 5, after 6 h, the *E. coli* maximum glucose consumption resulted in the lowest concentration of AZA (31.2 µg/mL). It is readily apparent that the increased AZA concentration avoids sugar consumption because of its inhibitory effect on bacterial CAs, which are directly involved in providing CO_2_/HCO_3_^−^ necessary for the bacterial metabolic demand.

## 4. Conclusions

In this article we focalise our attention on bacterial metabolic pathways in which bacterial CAs have a central role. These enzymes are thus attractive drug targets. To demonstrate their draggability, we used *E. coli* cells as a model system, since they can be easily manipulated in the lab, and used facile general methods for determining bacterial survival, bacterial growth and carbohydrate consumption. In pharmacological application, these assays are mostly used to evaluate the viability and compare the impact of various products on microbial cultures. As a result, we demonstrated that AZA, a sulphonamide FDA-approved CA inhibitor, impairs bacterial growth as well as the glucose uptake, which *E. coli* (and other bacteria) use as a carbon source for growth and metabolism. Intriguingly, the inhibitory effect of AZA on the *E. coli* culture became apparent at concentrations a slightly higher than 31.2 µg/mL. Thus, we demonstrate here that the AZA that inhibits *in vitro* the two bacterial CAs, in the nanomolar range, also inhibits *in vivo* the growth of the bacterium. The strategy used in the present work affords a proof-of-concept study and brings new insights into the choice of novel and efficient CAIs capable of impairing bacterial metabolism, especially in relation to the fact that such eventually newly found compounds might be potentially used to combat drug resistance of many pathogens to the existing antimicrobial drugs.
